# Material-Sensitive and Thickness-Resolved Transmission Imaging Using Coherent Extreme Ultraviolet Radiation

**DOI:** 10.1021/acsphotonics.5c01717

**Published:** 2025-11-13

**Authors:** Fengling Zhang, Xiaomeng Liu, Antonios Pelekanidis, Matthias Gouder, Kjeld S. E. Eikema, Stefan Witte

**Affiliations:** † 530573 Advanced Research Center for Nanolithography, Science Park 106, 1098 XG Amsterdam, The Netherlands; ‡ Department of Physics and Astronomy, 1190 Vrije Universiteit, De Boelelaan 1105, 1081 HV Amsterdam, The Netherlands; § Imaging Physics, Faculty of Applied Sciences, Delft University of Technology, Lorentzweg 1, 2628 CJ Delft, The Netherlands

**Keywords:** high-harmonic generation, extreme ultraviolet coherent diffractive imaging, computational imaging, diffractive shearing interferometry, ptychography

## Abstract

Microscopy with extreme ultraviolet (EUV) radiation enables high-resolution imaging with excellent material contrast because of the short wavelength and numerous element-specific absorption edges available in this spectral range. Table-top high-harmonic generation (HHG) sources offer the additional advantage of generating wide spectra in the EUV and soft X-ray range, making them inherently well-suited for characterizing nanostructures. As lens-based EUV imaging is challenging, lensless imaging methods based on coherent diffraction offer practical advantages and can even allow for quantitative phase measurements of object transmission functions. Here, spectrally resolved lensless imaging of a dispersive sample is performed using multiple high harmonics based on different HHG-based measurement concepts. We characterize the structure and composition of a three-element spiral-shaped object in transmission using multiwavelength diffractive shearing interferometry, as well as single-wavelength structured-illumination ptychography. We find that both methods are capable of retrieving spatially resolved element maps and the corresponding layer thicknesses. Comparing methods, ptychography provides superior accuracy in determining layer thickness, even for stacks of multiple materials, using an extended scattering quotient. These measurement and analysis concepts thus provide a nondestructive way to accurately extract information on the material composition and layer thicknesses of complex nanostructured samples.

## Graphical abstract

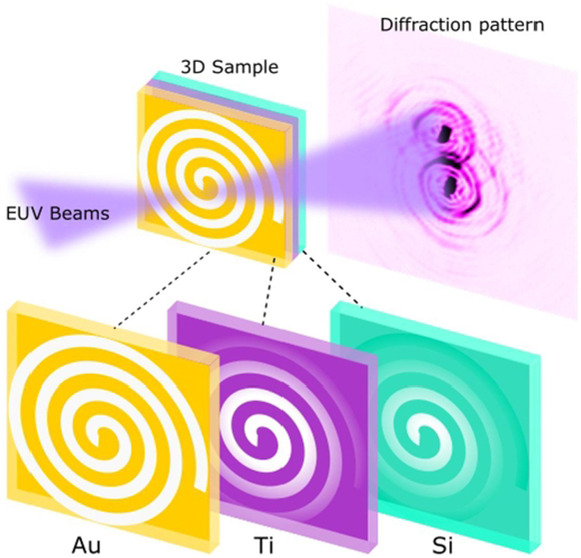

## Introduction: Material-Sensitive Coherent Diffractive Imaging with High Harmonic Sources

Breakthroughs in imaging are driving advances in nanoscale metrology, enabling more precise multiscale three-dimensional characterization of functional systems such as integrated circuits in the semiconductor industry. Unlike traditional microscopy, which requires wavelength-specific lenses for photons and electrons, coherent diffractive imaging (CDI) captures diffraction patterns directly and employs numerical phase-retrieval algorithms to reconstruct an object.^1–3^ The resulting object image can be numerically corrected for aberrations, with achievable resolution limited only by the wavelength of the incident radiation and the spatial frequency of the diffracted waves. To approach the diffraction limit, CDI and especially its scanning version known as ptychography^4^ have been extensively explored in the short wavelength spectral ranges such as EUV^5–11^ and X-ray,^12–14^ as well as for electron imaging.^15–17^

While CDI enables high-resolution imaging, its reconstruction quality depends on the signal-to-noise ratio of the diffraction patterns.^18,19^ Current coherent sources like synchrotron radiation and free-electron lasers fulfill the high brightness requirements, yet their accessibility limits the widespread implementation of CDI techniques.^13,14,20^ Meanwhile, table-top high harmonic generation (HHG) provides the possibility to generate a broad harmonic spectrum of spatially coherent EUV radiation with a laboratory-scale setup.^21^ In recent years, HHG sources have been widely applied in nanoscale coherent imaging from interferometry,^22,23^ reflectometry,^6,10^ and wavefront sensing^24–27^ to both material^7,28,29^ and biological science.^30^

For semiconductor applications, imaging and inspection with HHG sources present an attractive option, as many materials exhibit unique absorption and transmission properties in the EUV wavelength range.^31^ This capability makes HHG-based imaging an excellent tool for characterizing nanostructures. A first demonstration at 13.5 nm imaged the nearly periodic structure on a silicon-based zone plate with subwavelength spatial resolution of 12.6 nm with a corresponding relative height map.^32^ Recent ptychographic studies utilize information on both absorption and phase shift in every pixel of the sample image, enabling chemically resolved imaging by calculating the so-called scattering quotient.^7,29,33^ Reflection-mode ptychography enabled nondestructive determination of layer thicknesses with chemically specific contrast for substrate-based samples.^6,10,28^ For thin, transparent samples, having a transmission-based measurement that is sensitive to both the material composition and thickness of various layers would be particularly relevant.

To characterize layer thickness and material composition in transmission, both attenuation and phase shift upon propagation are relevant measurable quantities. The concept of scattering quotient is based on the ratio of these quantities^7^ but removes the sensitivity to layer thickness. Another approach can be to utilize the wavelength dependence of such material properties by performing, for example, intensity-only measurements at multiple wavelengths. In this work, we perform different HHG-based lensless imaging experiments aimed at retrieving element-resolved images of a multielement structured film in transmission. Specifically, we use both diffractive shearing interferometry (DSI)^22,23^ and ptychography and compare the ability of both methods to provide quantitative information on elemental composition and layer thicknesses. DSI is ideally suited for multiwavelength measurements, as it is based on Fourier-transform spectroscopy to retrieve spectrally resolved diffraction information. Ptychography is developed for single-wavelength measurements although it can be extended to multiwavelength reconstructions as well.^5,10,11,34^ An important advantage of ptychography is the ability to separate the probe beam information, leading to accurate quantitative phase retrieval and enabling imaging with structured probe beams.

By applying both methods to the same sample and comparing the results with energy-dispersive X-ray spectroscopy (EDX) from a scanning electron microscope (SEM), we analyze their respective performance. We find that especially the quantitative amplitude and phase information provided by ptychography allow for an accurate sample characterization. By extending the concept of the scattering quotient to include multiple layers of different materials, we can determine the local thickness of the two layers of distinct materials across the sample. We therefore conclude that ptychography is a useful nondestructive approach to characterize both material composition and layer thickness in complex nanostructured thin film samples.

## Coherent Diffractive Imaging Techniques

As image sensors record only the intensity, the main challenge in CDI is to retrieve the phase information belonging to a measured diffraction intensity profile. In the far-field limit, the diffracted electric field corresponds to the Fourier transform of the exit wave, which is typically modeled as the product of the illumination field (’probe’) and the complex object transmission function. To reconstruct the exit wave, the phase of the electric field at the detector plane is retrieved using iterative methods^2,35^ and subsequently numerically propagated to the object plane. The two specific implementations of CDI that we use are DSI and ptychography, which are schematically depicted in Figure 1 and are introduced in more detail below. To compare the main properties of both methods, Table 1 summarizes the advantages and current limitations of both DSI and ptychography.

**Figure 1:**
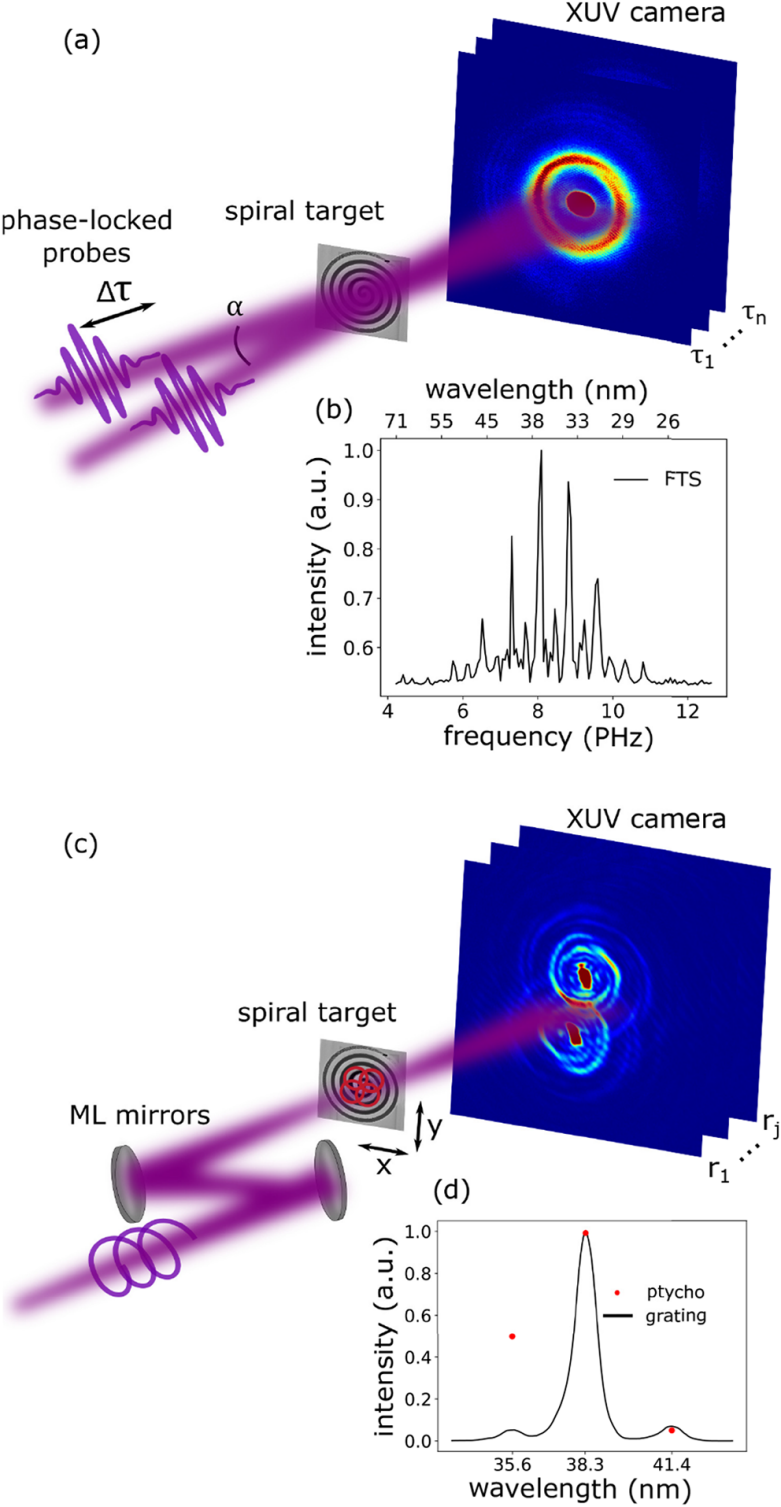
Schematic of the different CDI concepts used in this work. (a) Diffractive shearing interferometry: two identical and mutually coherent HHG beams illuminate the object (being a spiral target in our experiments) with a finite shear angle α between them and a controlled time delay Δ*τ*. A series of diffraction patterns is recorded as a function of τ, from which spectrally resolved diffraction patterns can be reconstructed.^36^ (b) Typical HHG spectrum retrieved from a time-delay scan without an object present in the beam. (c) Ptychography: the HHG beam is refocused and spectrally filtered by a pair of narrow-band multilayer mirrors onto the object mounted on a translation stage. A series of diffraction patterns are recorded as a function of transverse object position relative to the probe *r*_*j*_. In these experiments, and the EUV beam is spatially structured by imparting orbital angular momentum onto it in the HHG process. (d) Measured spectrum of the EUV radiation after the spectrally selective mirrors, along with spectral weights as retrieved by ptychography (see text for details).

**Table 1:** Comparison of DSI and Ptychography

method	FTS+DSI	ptychography
spectrally resolved	√no prior knowledge needed	√needs well-calibrated prior input
quantitative phase retrieval	×	√
amplitude retrieval	√	√
advantages	single-shot imaging; high spatial and temporal resolution	reconstructs probe and object; large field of view with nanoscale resolution; compensate partial coherence and imperfect measurements via a mixed-state model.
limitations	prior knowledge of sample; cannot separate phase contributions from object and probe.	prior knowledge of spectrum; high computational cost.

### Diffractive Shearing Interferometry

The DSI approach^22^ is based on the concept of spatially resolved Fourier transform spectroscopy (FTS)^37^ combined with lateral shearing interferometry.^38–40^ The experimental concept is shown in Figure 1a. The object is illuminated by a pair of broadband noncollinear HHG sources, which are produced using phase-locked pairs of intense driving laser pulses.^36,37^ The two beams illuminate the sample target at slightly different angles. An EUV-sensitive CCD camera positioned downstream captures the resulting diffraction pattern, which corresponds to the coherent sum of the two spatially displaced (sheared) exit waves in the far field. By scanning the time delay τ between the HHG pulses, a series of far-field diffraction patterns are recorded. The time delay scan gives rise to an interference pattern analogous to Fourier-transform spectroscopy at each CCD pixel, enabling the reconstruction of diffraction patterns for each wavelength present in the HHG illumination.^36^ For each wavelength component, the complex measured interference pattern at the camera plane can then be expressed as^22^

1 $$\begin{array}{l} M\left(k\right)=E\left(k+dk\right)E{\left(k-\mathrm{dk}\right)}^{*}\\ =A\left(k+dk\right)A\left(k-dk\right)\exp\left\{i\left(\phi \left(k+dk\right)-\phi \left(k-dk\right)\right)\right\} \end{array}$$

where *A*(*k* + d*k*) is the amplitude of the electric field *E*(*k* + d*k*) of one beam, ϕ­(*k*) is the phase of the electric field, and *k* is the k-space coordinate as recorded in the camera plane. Given the lateral shear between the two beams 2d*k*, a general camera-plane constraint for iterative phase retrieval can be written as

2 $$E_{n+1}\left(k\right)=\left(1-\beta \right)E_{n}\left(k\right)+\frac{\beta }{2}\left[\frac{M\left(k-dk\right)E_{n}\left(k-2dk\right)}{|E_{n}\left(k-2dk\right){|}^{2}+\epsilon^{2}}+\frac{M^{*}\left(k+dk\right)E_{n}\left(k+2dk\right)}{|E_{n}\left(k+2dk\right){|}^{2}+\epsilon^{2}}\right]$$

where *E*_*n*_ is the *n* ^*th*^ guess of the electric field, *β* is the strength of the correction to the electric field guess and is typically set to 0.9, and ϵ is a small number to avoid zero divisions. In combination with prior knowledge of object support, the monochromatic electric field can be acquired by using different phase-retrieval algorithms.^1,35,41^

An advantage of DSI is that the complex phase term provides information about the spatial phase derivative along the direction of shear between the beams. Furthermore, information at multiple wavelengths is recorded in parallel, making effective use of the available HHG flux and bandwidth and avoiding the systematic errors involved in wavelength-scanning measurements. During DSI, the use of near-plane-wave illumination ensures that the diffraction pattern remains unchanged with the sample, greatly simplifying the sample alignment during reconstruction. However, the retrieved phase information still contains the wavefront of the illumination beam, making it challenging to unambiguously isolate the sample-induced phase shifts from the inherent phase curvature of the probe.

### Ptychography

As shown in Figure 1c, ptychography utilizes a spatially confined probe beam that is transversely scanned across the sample with partial overlap between adjacent positions.^4^ This scanning strategy imposes strong constraints on possible exit wave solutions, leading to robust and accurate phase retrieval. As ptychography can be extended to separately retrieve the complex fields of both probe beam and the sample response,^42^ the illumination profile does not need to be accurately known a *priori*. This allows the use of structured illumination to improve reconstruction quality.^9,11^ We perform our ptychography reconstructions with the package PtyLab.py,^43^ which includes the ability for ptychographic information multiplexing.^34^ Here, the measured far-field diffraction pattern at position *j* is described as the incoherent sum of the *k* monochromatic diffraction patterns. For our experiments on a dispersive sample, we modify the forward model to include a wavelength-dependent object transmissivity

3 $$I_{j}∼\sum_{\lambda }\sum_{k\in \left\{0,1\right\}}{\left({\mathrm{P̂}}_{\lambda }\left[P_{k,\lambda }\left(r\right)\cdot O_{\lambda }\left(r-r_{j}\right)\right]\right)}^{2}+I_{B}$$

where *λ* denotes the wavelength and *k* refers to the orthogonal modes in the decomposition of the mutual intensity of a spatially partially coherent beam.^7,11,44^ Both object *O* and probe *P* are modeled in terms of a set of modes covering the different harmonic wavelengths. Additionally, **P̂**_λ_ is the scaled angular spectrum propagator,^18,45,46^ which allows the propagation of an electromagnetic wave under the Fresnel approximation, maintaining a wavelength-independent pixel size at the object plane. Although we use a narrow-band multilayer mirror in the experiment, the mirror pair still reflects an additional harmonic on each side of the central harmonic, as shown in Figure 1d. *I*_*B*_ is a constant background caused by leakage of the fundamental beam to the detector and thermal effects from the detector itself.

Ptychographic methods remove the need for any object support and the associated prior object knowledge.^4^ However, in addition to the computational complexity, ptychography relies on accurate knowledge of the wavelengths and the distance between the object and the detector plane to ensure the scaled angular spectrum propagator **P̂**_λ_ can be applied accurately although such parameters can be optimized numerically to a certain extent.^47^

## Materials and Methods

### Nanofabrication of a Dispersive Multilayer Sample

To test the performance of the different CDI methods on a dispersive and multielement sample in transmission, we fabricated a dedicated test object. Such a partially transparent sample was fabricated by using a combination of sputter coating and focused ion beam (FIB) milling techniques. A 50 nm thick freestanding Si_3_N_4_ membrane was used as the substrate, coated with a 59 nm thick layer of titanium, followed by 100 nm thick gold capping layer. As shown in Figure 2b, a spiral-shaped pattern is milled onto the multilayer substrate using the FIB processing. The narrow-line width spiral structure generates far-field diffraction patterns with strong high-angle components, providing rich spatial frequency information that benefits CDI reconstruction.

**Figure 2:**
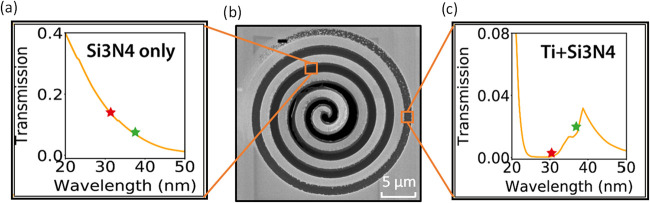
Transmission as a function of wavelength for the (a) Si_3_N_4_ layer and (c) Si_3_N_4_+Ti layer, respectively. The red star indicates the transmission at 31 nm, while the green star indicates transmission at 37 nm. (b) SEM image of the spiral target.

A key feature of the design is the spiral pattern’s varying depth profile, which gradually changes from the center outward. At the very center, all layers are fully removed, resulting in a full transmission. As the spiral line progresses toward the outside, the Si_3_N_4_ layer starts to appear and gradually increases in thickness to 50 nm. The EUV transmission profile for a 50 nm Si_3_N_4_ layer is shown in Figure 2a. Further toward the outside of the spiral, the Ti layer also remains present with gradually increasing thickness. The EUV transmission for 50 nm Si_3_N_4_ plus 59 nm Ti is shown in Figure 2c. At the tail of the spiral, the full thickness is retained. In this region, the transmission is expected to drop to zero, as the gold layer is fully opaque for our HHG spectrum. To mark where the spiral ends, a fully transmissive rectangular hole is milled as a reference marker. As the Si_3_N_4_ and Ti layers have different spectral responses and especially Ti has an absorption edge leading to a strong reduction in transmissivity at wavelengths below 30 nm, the resulting object is expected to have a strong spatially dependent spectral response in both amplitude and phase.

### Experiment Design

The DSI experimental setup is shown in Figure 1a. The two EUV beams intersect at a relative angle α of 0.4 mrad, resulting in two sheared copies of the diffraction pattern on the CCD camera placed 18 cm downstream from the sample plane. The CCD chip has 2048 × 2048 pixels with 13.5 μm pixel size. The sample transmissivity was low, which necessitated an 18 s integration time for each camera exposure with 4-by-4 binning and 4× camera preamplifier gain. The time-delay scan was optimized for the HHG spectrum in argon gas jet, with a time delay step of approximately 32 as, corresponding to 9.7 nm optical path difference, enabling measurement of the theoretically shortest wavelength of 19.4 nm. The entire experiment comprised 485 time delay steps, resulting in a total delay of 15.7 fs, spanning multiple optical cycles of the driving laser, and ensuring sufficient spectral resolution.

The ptychographic experimental setup is shown in Figure 1c. The fundamental laser driving the HHG process contained orbital angular momentum (OAM) with charge one, leading to harmonics carrying OAM as well.^48^ This nonzero OAM results in a significantly structured illumination profile of the probe, which improves the reconstruction quality and algorithm convergence.^9,11^ The structured high harmonics are refocused by a pair of narrow-band multilayer mirrors onto the sample. The CCD camera is placed approximately 10.68 cm from the sample plane. A typical multispectral HHG diffraction pattern corresponding to illumination of the central area of the sample is shown in Figure 1c. The ptychographic data sets for the OAM beams consist of 301 scan positions in a concentric scan grid with a 3.76 μm step size and 67 μm field of view. The EUV spectrum is measured separately by placing a transmission grating with 500 nm pitch (solid line in Figure 1d) in the HHG beam. Furthermore, ptychography retrieves the spectral weights of the different probe modes,^25^ allowing a comparison (Figure 1d). The multilayer mirror pair is specifically designed to reflect the 27th harmonic (38.3 nm), while the neighboring 25th and 29th harmonics exhibit significantly lower signal strengths, approximately 10% of that of the 27th harmonic. A discrepancy between the retrieved spectrum and grating measurement is observed at the 25th harmonic at 35.6 nm, where the signal-to-noise ratio is the lowest. Therefore, we focus on the ptychographic results at 38.3 nm in the rest of this paper.

## Results and Discussion

### Qualitative, Material-Resolved DSI Results

Figure 3(a,b) shows the retrieved monochromatic diffraction patterns at wavelengths of 31 nm (red trace) and 37 nm (green trace) resulting from the time-delay scan, respectively. The wavelength scaling between the patterns at 31 and 37 nm is clearly visible. The DSI iterative phase-retrieval method is employed to reconstruct the spiral images at both 31 and 37 nm wavelengths from their corresponding diffraction patterns.

**Figure 3:**
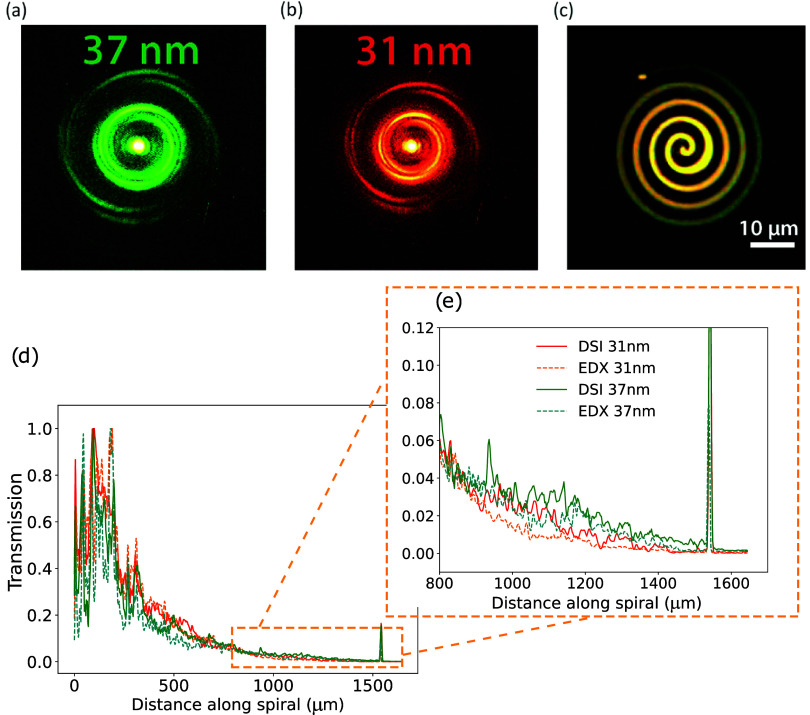
DSI measurement results. Monochromatic diffraction patterns at a wavelength of (a) 37 nm and (b) 31 nm. (c) Image reconstructions superimposed at wavelengths of 37 nm (green) and 31 nm (red). A yellow color represents similar transmission at both wavelengths, while red or green color indicates higher transmission at 31 or 37 nm, respectively. (d) Comparison of the relative smooth transmissivity along the spiral, as determined by DSI at 31 nm (red solid line) and 37 nm (green solid line) wavelength and calculated from the layer thicknesses determined from EDX (see text for details) data for 31 nm (orange dashed line) and 37 nm (blue dashed line), respectively. (e) Zoomed-in version of (d) for the outer part of the spiral.

The reconstructed spiral transmissivity images at 31 and 37 nm are visualized using color mapping, with red representing 31 nm and green representing 37 nm. The two reconstructed spiral images are superimposed, as illustrated in Figure 3c, with their transmissivities normalized at the center of the spiral where no material is present. The color in the composite image reflects the transmission strength at the two wavelengths. In the central region, a yellow hue indicates comparable transmission at 31 and 37 nm. A gradual shift in color is observed toward red in regions primarily composed of Si_3_N_4_ and toward green in regions containing Ti. These trends meet good agreement with the expected transmission properties of the respective materials shown in Figure 2.

The DSI data are analyzed by taking a line-out along the spiral line to determine the experimentally observed normalized transmissivity along the spiral. As an independent reference, we performed EDX measurements on the same sample and calculated the expected sample transmissivity from the observed local elemental composition. More details on the EDX data and analysis are given in Supporting Information S1 and S2. The comparison of the DSI and EDX results is shown in Figure 3d. The reconstructed transmissivity from DSI (solid traces) is consistent with the EDX results (dashed traces). Figure 3e shows the transmissivity across the outer ring of the spiral structure, where a larger amount of Ti remains on the sample. Significantly, the transmissivity at 37 nm is higher than that at 31 nm in both DSI and EDX data sets. This wavelength-dependent variation in transmissivity suggests the presence of both Si_3_N_4_ and Ti in the region between around 500 and 1500 μm distance along the spiral path.

DSI measurements provide an accurate spectrally resolved imaging method. Qualitatively, good agreement is found between the transmission percentages extracted from the DSI data and those from the EDX data. However, accurately extracting quantitative layer thicknesses solely on the basis of the amplitude information from DSI is found to be challenging. Although the DSI and EDX data show consistent trends at both 31 and 37 nm, the level of noise in the DSI-derived transmissivity is comparable to the wavelength-dependent signal differences, limiting the precision of any layer thickness estimation. To achieve more accurate material-specific and elemental characterization, the phase of the exit wave can be taken into consideration, which allows mapping of the spatial variations in optical path length across the sample. In DSI reconstruction, however, the measured phase contains contributions from both the sample and the illumination beam, and separating the phase shift introduced by the dispersive sample from the inherent phase curvature of the probe is a significant challenge. Therefore, any quantitative analysis of DSI image reconstructions typically remains limited to intensity information. The following section explores how ptychography offers a promising approach to overcome this limitation.

### Quantitative, Material-Resolved and Thickness-Sensitive Ptychography Measurements

The results of the ptychography measurements are summarized in Figure 4. The reconstructed spiral target is shown in Figure 4a. Given the current experimental parameters, the diffraction-limited resolution is 138 nm, assuming the shortest contributing wavelength component is 35.6 nm. Since the reconstructions give complex-valued expressions for both the object and the probe, numerical propagation was applied to the object to correct for a defocus term arising from calibration mismatches in the wavelength or the sample-to-camera distance. Figure 4b shows the reconstructed probe, which is 27th harmonic generated from our home-built high-power laser operating at 1030 nm central wavelength.^49^ Two incoherent probe modes at 38.3 nm are reconstructed, expressed by the orthogonal probe modes in eq 3. The main mode *k* = 0 contains 62% of the intensity, with an overall mode purity of 71.2%.^44^ Given that HHG sources typically exhibit a high degree of spatial coherence,^50^ we attribute the mixed-state modes to partial decoherence and other sources of noise in the forward model. The reconstructed probe does not exhibit the typical donut-shaped profile characteristic of standard OAM beams. This deviation is primarily attributed to strong astigmatism introduced by the multilayer mirrors, as discussed in detail in our previous work.^27^

**Figure 4:**
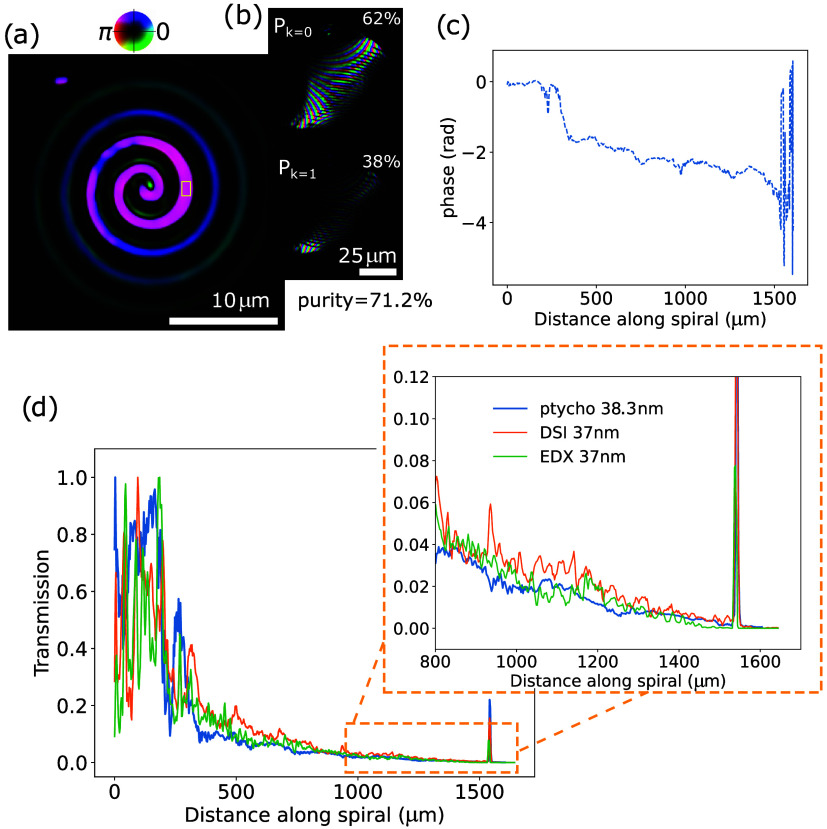
Ptychography reconstructions of (a) the spiral sample and (b) the main spatial modes of the OAM beam at 38.3 nm. Here, the amplitude and phase are represented by brightness and color, respectively. (c) Extracted unwrapped phase along the trajectory of the spiral. (d) Comparison of the relative transmissivity along the spiral, as determined by ptychography at 38.3 nm (blue trace), DSI at 37 nm (orange trace), and EDX at 37 nm (green trace) (DSI results at 37 nm wavelength corresponds to the 23rd harmonic of a Ti/Sa-driven HHG source. Ptychography results at 38.3 nm corresponds to the 27th harmonic of the Yb/YAG-driven HHG source.).

Similar to the DSI analysis, we retrieved the transmissivity along the spiral line. As ptychography separates the complex fields of the object and probe, it becomes possible to quantitatively assess the phase delay introduced by the object along the spiral trajectory as well (Figure 4c). From the retrieved transmission intensity, we find that the transmission curve agrees well with the DSI and EDX measurements (Figure 4d). The root-mean-square (RMS) deviations between DSI-EDX and ptychography-EDX are calculated over the region from 500 to 1500 μm, where both Si_3_N_4_ and Ti are present, giving values of 0.02 and 0.01, respectively.

Knowing the phase profiles of the spiral sample enables us to analyze its material properties in more detail. Similar to the intensity, there is a distinct jump in the accumulated phase around 250 μm along the spiral, which has much better visibility in the phase profile compared to the noisier intensity profile. Further along the spiral path, the phase delay continues to increase monotonously, as expected for an increasing amount of material but with a varying slope. This behavior indicates that the spiral sample exhibits a more complex structure than a simple material with a linearly increasing thickness. Given the limited control over the rate of material removal in the FIB milling process, especially in the presence of multiple elements, a linear thickness profile would not necessarily be expected for the fabricated spiral path.

For a more quantitative analysis of the local material composition, we use the retrieved amplitude and phase images to calculate the scattering quotient averaged along the projection direction (Figure 5a). The scattering quotient allows the identification of different materials by comparison with the measured complex refractive indices of different materials.^7,30^ With the complex refractive index given as *n* = 1 – δ – *iβ*, the scattering quotient is defined as the ratio *f*_*q*_ = δ/β, which can be shown^7^ to be equivalent to the ratio of the measured phase delay and the logarithm of the object transmissivity amplitude

4 $$f_{q}=\frac{\phi \left(x,y\right)}{\ln\left(|A\left(x,y\right)|\right)}=\frac{\delta }{\beta }$$

An advantage of such a scattering quotient is that it is independent of the layer thickness, making it a sensitive probe to identify materials in the case of a single element at each location. In the case where multiple elements are stacked, a similar approach can still be used to connect the measured amplitude and phase to the local material properties. We define an extended scattering quotient, which for a two-layer system takes the following form

5 $$f_{q}=\frac{\phi \left(x,y\right)}{\ln\left(|A\left(x,y\right)|\right)}=\frac{\delta_{1}+\delta_{2}\left(d_{2}/d_{1}\right)}{\beta_{1}+\beta_{2}\left(d_{2}/d_{1}\right)}$$

where the refractive index of the Si_3_N_4_ is given by *n*_1_ = 1 – δ_1_ – *iβ*_1_ and the subscript ‘2’ refers to Ti. A derivation of this extended scattering quotient is provided in Supporting Information S3, and a further extension to more than two materials is straightforward. For locations on the sample where the Ti layer is removed (i.e., *d*_2_ = 0), *f*_*q*_ only corresponds to Si_3_N_4_. eq 5 then reduces to the form for a single material (eq 4 and again becomes independent of material thickness).

**Figure 5:**
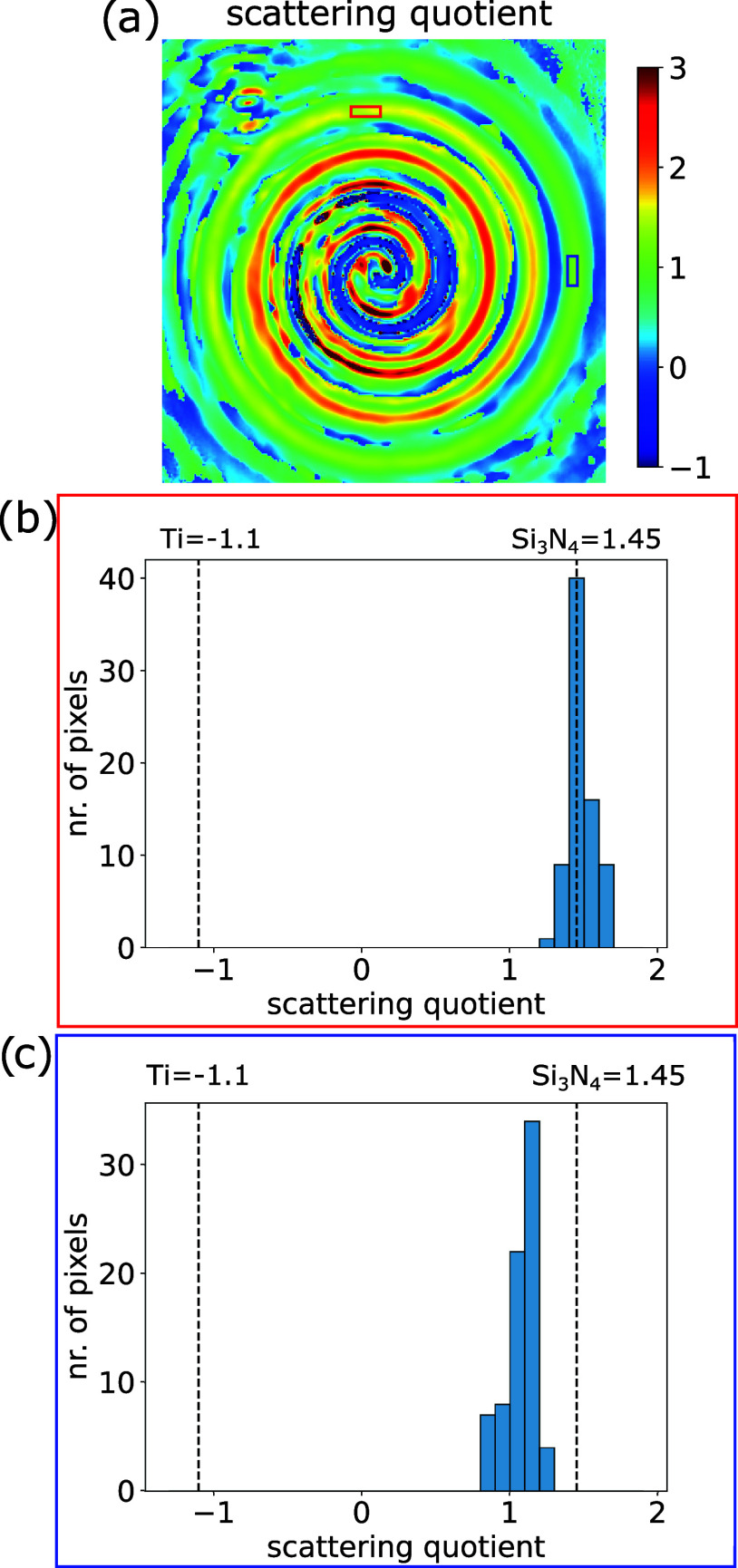
(a) Scattering quotient map from the reconstructed spiral sample. (b) and (c) Histograms representing the data from the selected regions (blue and red squares in (a), respectively).

To determine the spatially resolved scattering quotient across the object, we take an area at a position where the spiral is fully open (indicated by the yellow rectangle in Figure 4a) as a reference. The object transmissivity amplitude *A*(*x*, *y*) and phase delay ϕ­(*x*, *y*) are then determined with respect to that reference and used to calculate the scattering quotient. The result is shown in Figure 5a. Along the spiral path, a continuous change in the scattering quotient is observed, indicating a change in the material composition. For further analysis, we plot histograms of the scattering quotient per pixel across two selected regions located in different rings indicated by the red and blue areas in Figure 5a, respectively. In the histogram of the red region (Figure 5b), corresponding to the inner ring, the scattering quotient peaks around 1.45 at 38.3 nm. This value is calculated by eq 4 for Si_3_N_4_,^51^ *f*_*q*(*Si*_3_ *N*_4_)_ = δ/β = 0.245/0.168 = 1.45, indicating that there is a single material present in this area. In contrast, the histogram of the blue area in the outer ring (Figure 5c) shows a significantly lower scattering quotient, which can be attributed to the presence of Ti on top of the Si_3_N_4_ membrane. As expected from eq 5, a value for the scattering quotient is found that effectively is a weighted average of the values of the separate elements with the relative thickness of the two layers as the weighting factor.

For a sample consisting of two materials, it is possible to determine the thicknesses of both layers from the reconstructed amplitude and phase delay. Assuming weakly reflecting layers and linear propagation,^51^ the thickness of the two layers can be expressed as follows: for a sample consisting of two materials, it is possible to determine the thickness of both layers from the reconstructed amplitude and phase delay. Assuming weakly reflecting layers and linear propagation,^51^ the thickness of the two layers can be expressed as

6 $$d_{1}=\frac{C_{1}\delta_{2}-C_{2}\beta_{2}}{\beta_{1}\delta_{2}-\beta_{2}\delta_{1}}$$

7 $$d_{2}=\frac{C_{2}\beta_{1}-C_{1}\delta_{1}}{\beta_{1}\delta_{2}-\beta_{2}\delta_{1}}$$

Here, *C*_1_ = – (λ_0_/2π) ln­(|*A*|) and *C*_2_ = (λ_0_/2π) ϕ. A derivation of these expressions, along with an error analysis of the thickness determination, is given in Supporting Information S4. Figure 6 shows the results of such a layer thickness analysis on our spiral target. The achieved 539 nm spatial resolution, shown in Figure 6d, is determined using Fourier ring correlation (FRC)^52^ between the high-resolution SEM image (Figure 2b) and the object reconstruction (Figure 6a). We average the measured amplitude and phase over a series of 5 × 5 *pixels* ^2^ areas (954 in total), indicated by white squares in Figure 6a, and calculate the thickness of the Si_3_N_4_ and Ti layers using eqs 6, 7. Starting from the spiral center to the outside, the thickness of both layers remains consistent with zero up to 150 μm. From there onward, a rapid increase in thickness of especially the Si_3_N_4_ layer is observed from 170 to 300 μm, as highlighted by the green dashed box in Figure 6e. This region corresponds to the green-marked areas in Figure 6a, where material removal appears to be inhomogeneous. After 300 μm, the Si_3_N_4_ thickness continues to increase at a much lower rate, while the thickness of the Ti layer starts to increase more rapidly. The marker at the end of the spiral is clearly visible at approximately 1500 μm, where the thickness of all of the materials drops to nearly zero. This region corresponds to the red-marked areas in Figure 6a. The nominal thicknesses of the layers are 50 nm for Si_3_N_4_ and 59 nm for Ti although these values can have a significant error margin from the fabrication tolerances.

**Figure 6:**
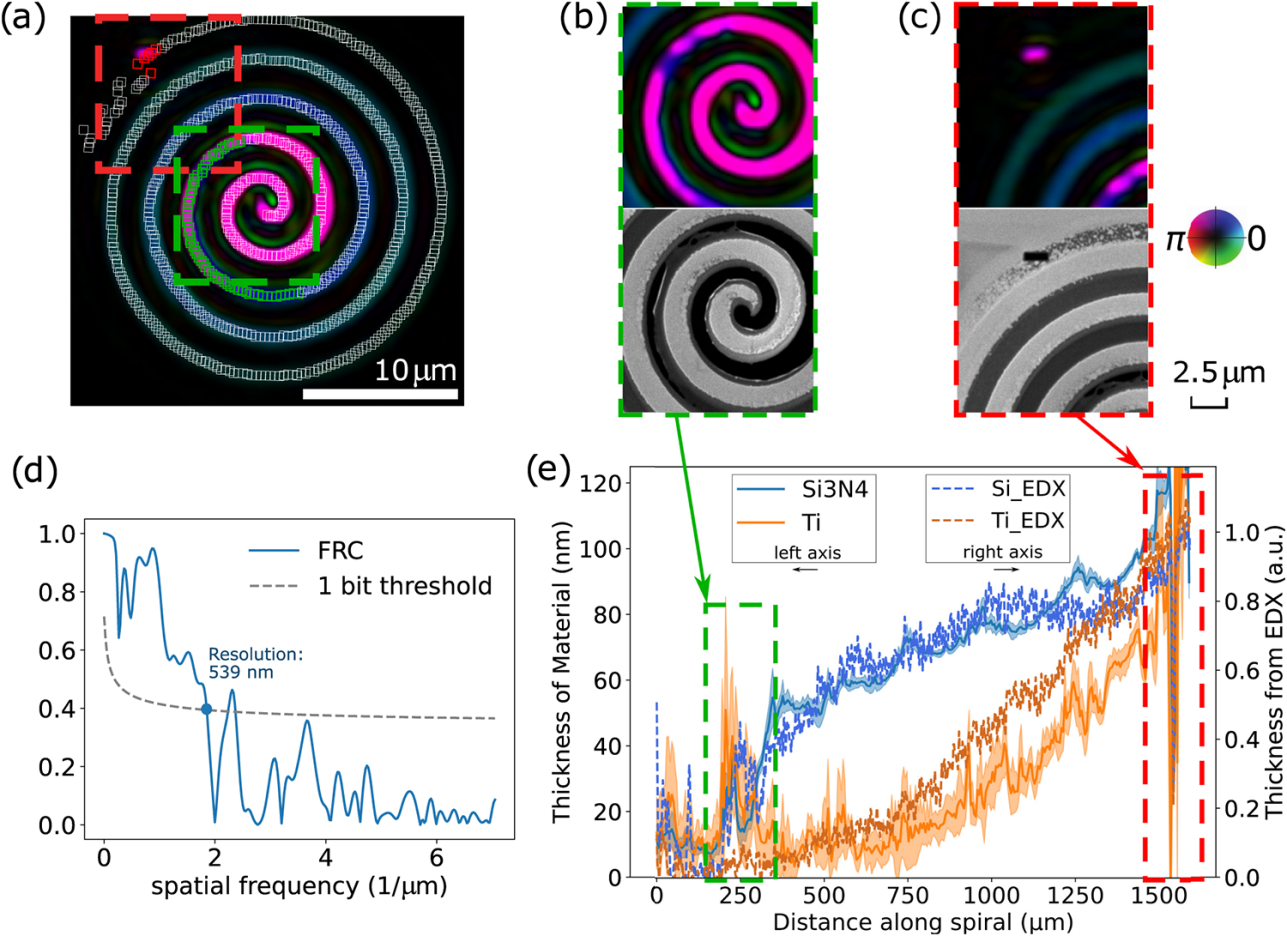
Quantitative thickness determination from ptychography reconstructions. (a) White squares indicate the regions selected along the line-out of the reconstructed complex transmissivity of the spiral sample. Regions of specific interest, highlighted in green and red in (a), are shown in detail in (b) and (c), respectively. (d) Fourier ring correlation (FRC) computed by comparing object reconstruction with object from SEM. (e) Absolute layer thicknesses extracted from the ptychography, including a comparison of the normalized layer thickness along the spiral sample as determined by EDX (dashed traces).

In Figure 6e, we also compare the material thickness retrieved from ptychography with an estimate of the EDX data. Although EDX cannot provide direct thickness values, for thin homogeneous layers, the relative signal strength can be interpreted as the total amount of material in a column and is therefore proportional to height (see Supporting Information S1). By normalizing the thickness to the ptychography value at the end of the spiral path, the shape of the EDX curve can be used for comparison. The center of the spiral target is completely fibbed through, resulting in no material remaining in that region. The thickness determination from EDX data is consistent with zero in this range, taking uncertainties and noise into account. These normalized curves show very good agreement, indicating that our ptychography-based approach can accurately characterize both material composition and layer thickness.

Aside from the overall good agreement, two regions along the spiral path show more complex behavior. The first such region is around 200 μm, which is where the Si_3_N_4_ film becomes visible. In this area, a large peak appears in the Ti thickness as well although with large error bars. This behavior seems to result from the rapidly varying spatial structure of the Si_3_N_4_ film, as can be seen in the SEM image in Figure 6b. Instead of a homogeneous layer, the film has various holes and curled remnants, which lead to deviations in the area-averaged scattering quotient. A second region with more complex behavior is toward the end of the spiral. Here, a deviation between the EDX estimate and the ptychography reconstruction is visible, with an overestimation of the Si_3_N_4_ thickness and an underestimation of Ti. These discrepancies are likely due to the presence of gold particle remnants on the spiral path, as can be seen in Figure 6c. The reference aperture at the end of the path is retrieved well, and the local layer thickness estimate is consistent with zero.

## Outlook and Conclusion

In this work, we performed HHG-based lensless imaging experiments aimed at retrieving element-resolved and thickness-sensitive information from a multilayer, compositionally complex nanostructured sample in transmission. DSI provides spectrally resolved reconstructions at 31 and 37 nm wavelengths. The method enables qualitative identification of regions dominated by Si_3_N_4_ and Ti across the sample based on their wavelength-dependent transmission properties. However, the ability of DSI to retrieve quantitative phase information was fundamentally constrained by the coupling of the object-induced phase with the curvature of the illumination beam. As a result, while it offers efficient multiwavelength imaging, DSI is limited in its capacity for precise thickness determination. Ptychography overcomes the limitations of DSI by reconstructing both the amplitude and the phase of the exit wave with a given spectrum. Using structured illumination with the OAM beams, we obtain high-quality reconstructions at a wavelength of 38.3 nm, enabling detailed analysis of the material composition and layer thicknesses of the sample. The scattering quotient method further facilitated material identification, while the combined amplitude and phase data allowed for accurate thickness extraction, validated by EDX. It is worth noting that the ptychographic results not only provided absolute layer thickness measurements but also revealed fabrication imperfections in specific regions, such as incomplete ion milling and residual gold particles, further highlighting the sensitivity and diagnostic potential of the method.

Combining these methods with multiwavelength ptychography would further increase the diagnostic capabilities, potentially giving sufficient information to extract thickness information from more than two layers (assuming refractive index information is available). Future work could combine DSI and ptychography to a multiwavelength region, in which a pair of HHG pulses with varying time delay is used in a ptychography scan.^53^ This scheme would combine increased wavelength diversity with multiwavelength amplitude and phase reconstructions of a complex object.

In conclusion, we find that ptychography provides a nondestructive framework for quantitative lensless EUV imaging. The ability to perform high-resolution, material-sensitive, and thickness-resolved analysis of complex nanostructures is promising for applications in nanolithography, semiconductor inspection, and multilayer thin film analysis, where access to buried structures and identification of material contrast is essential.

## Supplementary Material



## Data Availability

All data underlying the results of this paper may be obtained from the authors upon reasonable request.

## Notes

(I) Energy-dispersive X-ray spectroscopy results; (II) data analysis to raw transmissivity from DSI and EDX; (III) expression for the extended scattering quotient; (IV) thickness determination and error analysis (PDF)
