# Supplementary material for: Material-Sensitive and Thickness-Resolved Transmission Imaging Using Coherent Extreme Ultraviolet Radiation
Source: ACS Photonics. 2025 Nov 13;12(12):6680–9. doi: 10.1021/acsphotonics.5c01717 (PMC12715835; doi:10.1021/acsphotonics.5c01717)
Supplement: Supplementary file 1 [file ph5c01717_si_001.pdf]

Material-sensitive and thickness-resolved transmission imaging using coherent extreme ultraviolet radiation - Supplementary Information

Fengling Zhang,[†] Xiaomeng Liu,[†] Antonios Pelekanidis,[†] Matthias Gouder,[†] Kjeld Eikema,[†] and Stefan Witte[*,†,‡,¶]

†*Advanced Research Center for Nanolithography, Science Park 106, 1098 XG, Amsterdam, The Netherlands*

‡*Department of Physics and Astronomy, Vrije Universiteit, De Boelelaan 1105, 1081 HV Amsterdam, The Netherlands*

¶*Imaging Physics, Faculty of Applied Sciences, Delft University of Technology, Lorentzweg 1, 2628 CJ Delft, The Netherlands*

E-mail: smwitte@tudelft.nl

Energy-dispersive X-ray Spectroscopy Results

To establish a correlation between the reconstructed image and the chemical composition of the spiral sample, energy-dispersive X-ray spectroscopy (EDX) measurements were carried out, and the results are presented in Figs. SI1a-c), for the elements Si, Ti and Au, respectively. An EDX image encodes the abundance of the corresponding element in the brightness of the image. The Si image is representative of the Si_3N_4 layer, and appears dark in the center of the spiral, indicating that the layer has been completely removed. Along the spiral line, the brightness increases, indicating an increase in the thickness of the layer. Similar observations

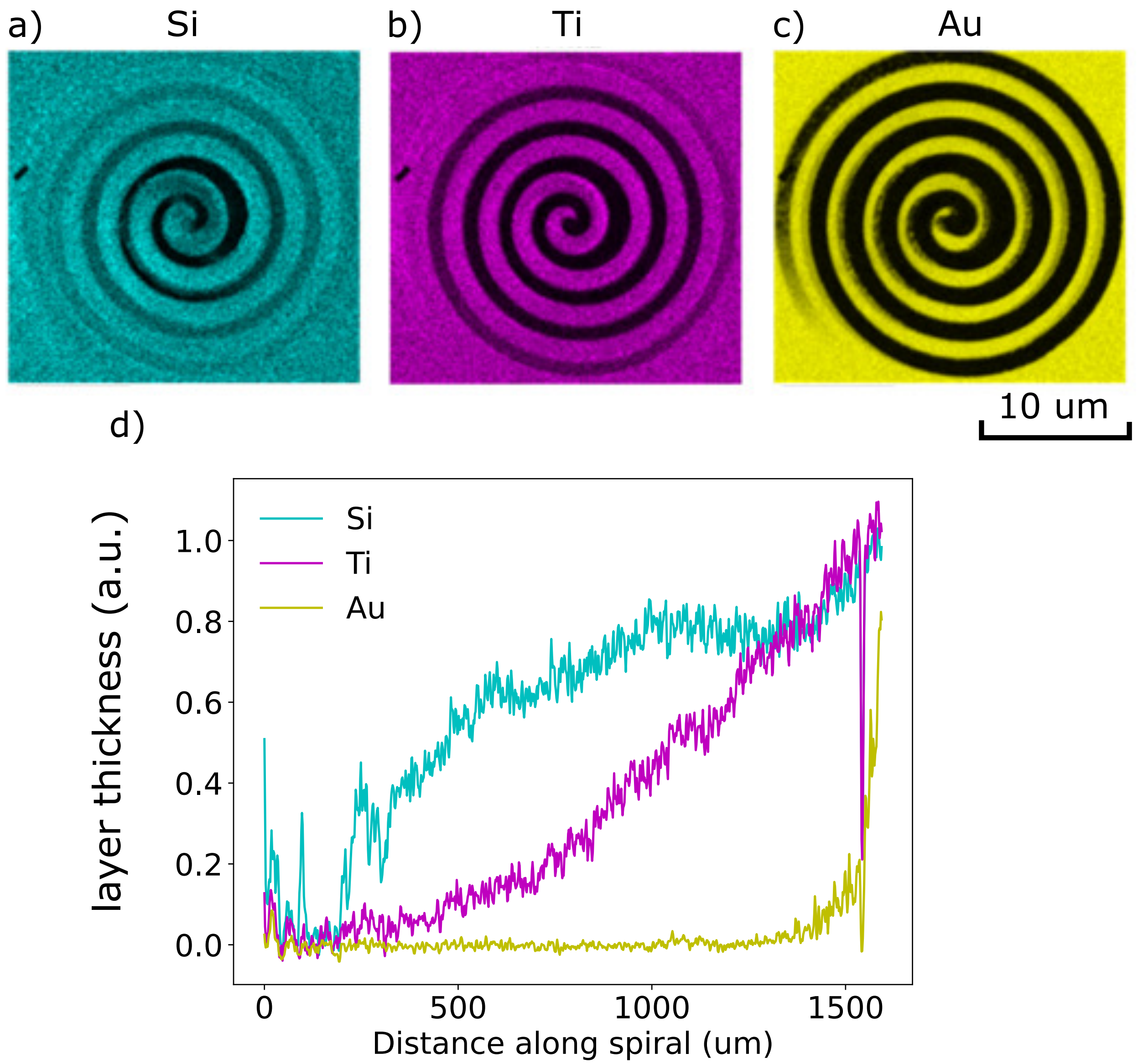

Figure 1: a-c) Energy-dispersive X-ray spectroscopy (EDX) measurements of the spiral sample: Si, Ti and Au data, respectively. d) Line-out along the spiral on the EDX data, giving an indication of the local layer thickness for each element.

were made for the other two elements, but with different gradients and positions where the material is fully removed. The EDX data is analyzed by taking a line-out along the spiral line and normalizing it to the signal outside the spiral, as illustrated in Fig 1b). This signal is interpreted as the local layer thickness d_l for each element. From this data, the EUV spectral transmission of each material is subsequently estimated through the utilization of a comprehensive X-ray database, provided by the Center for X-Ray Optics.[1] The Beer-Lambert

Law is then used to derive the transmission as a function of layer thickness, following the expression: $T_{layer} = T_t^{d_l/d_t}$, where T_t and d_t are the expected transmission and the nominal thickness of the fabricated layers. Finally, the overall transmission of the spiral thin-film is calculated by multiplying the individual layer transmissions.

Data analysis to raw transmissivity from DSI and EDX

Figure 2 shows the raw transmissivity data from DSI and EDX measurements. To improve visibility and signal-to-noise ratio, we have applied a smoothing procedure to the data, which leads to the final results shown in Fig. 3d-e) in the main text. For this smoothing, we convolute the raw transmissivity profiles with a rectangular window function of 3 μm width. While this does lead to a reduction of the spatial resolution along the spiral path, the noise reduction makes the resulting curves more readily comparable.

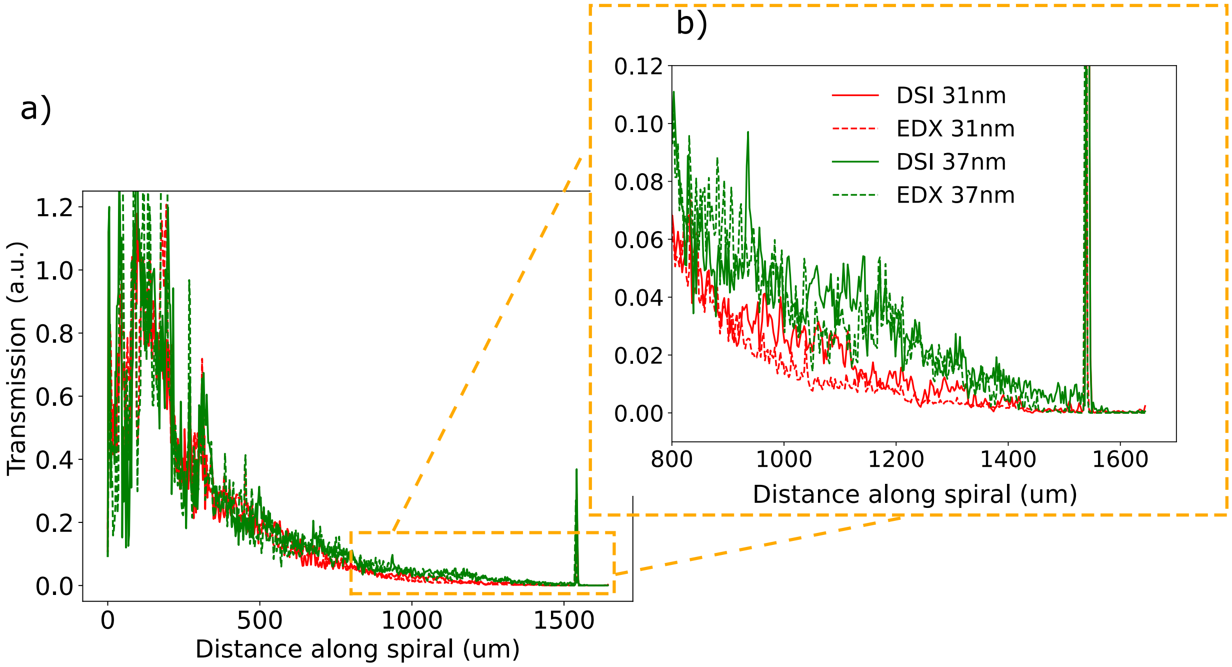

Figure 2: a) Comparison of the relative transmissivity along the spiral, as determined by DSI at 31 nm (red solid line) and 37 nm (green solid line) wavelength, and calculated from the layer thicknesses determined by EDX data for 31 nm (red dashed line) and 37 nm (green dashed line), respectively. b) Zoomed-in version of a) for the outer part of the spiral.

Expression for the extended scattering quotient

The scattering quotient can be applied to the case of multiple materials along the projection direction of the measurement. Starting from an incident field with amplitude E_0, the transmitted field behind the two-layer sample can be described by:

$$E(d_1, d_2) = E_0 \exp(-ik_1 d_1) \exp(-ik_2 d_2) \tag{SI. 1}$$

where d_1 and d_2 are the thicknesses of two different materials, respectively. The wavenumber $k_j = 2\pi n_j/\lambda$, with the refractive index of the material $n_j = 1 - \delta_j - i\beta_j$ and wavelength in vacuum λ_0. By inserting those parameters in Eq.SI. 1, the transmitted field can be written as:

$$E(d_1, d_2) = E_0 \exp(-\frac{2\pi}{\lambda_0}(\beta_1 d_1 + \beta_2 d_2)) \exp(-i\frac{2\pi}{\lambda_0}(\delta_1 d_1 + \delta_2 d_2)) \tag{SI. 2}$$

where the phase shift is taken relative to propagation in vacuum. From Eq. SI. 2 it directly follows that:

$$\ln A = \ln |E/E_0| = -\frac{2\pi}{\lambda_0}(\beta_1 d_1 + \beta_2 d_2)) \tag{SI. 3}$$

$$\phi = \frac{2\pi}{\lambda_0}(\delta_1 d_1 + \delta_2 d_2) \tag{SI. 4}$$

which can be combined and rearranged to give the expression for the extended scattering quotient in the main text, Eq. 5. The amplitude $|A| = |E/E_0|$ and phase of the electric field are both retrieved experimentally, allowing the calculation of the scattering quotient $f_q = \phi(x, y)/\ln(|A(x, y)|)$.

Thickness determination and error analysis

We rewrite Eqs. SI. 3, SI. 4 to separate the thickness terms:

$$\beta_1 d_1 + \beta_2 d_2 = -\frac{\lambda_0}{2\pi}\ln(|A|) = C_1 \tag{SI. 5}$$

$$\delta_1 d_1 + \delta_2 d_2 = \frac{\lambda_0}{2\pi}\phi = C_2 \tag{SI. 6}$$

From these equations, it is straightforward to isolate expressions for d_1 and d_2 in terms of the material constants, the wavelength and the measured amplitude and phase delay, given by Eqs. 6 and 7 in the main text.

An estimate of the accuracy of the thicknesses is given by an error analysis based on the uncertainties in the experimental parameters A and ϕ, which are included in C_1 and C_2. The standard deviation for thickness d_1 can be written as:

$$\sigma_{d_1} = \sqrt{\left(\frac{\partial d_1}{\partial C_1}\frac{\partial C_1}{\partial A}\sigma_A\right)^2 + \left(\frac{\partial d_1}{\partial C_2}\frac{\partial C_2}{\partial \phi}\sigma_\phi\right)^2} \tag{SI. 7}$$

where:

$$\begin{aligned} \frac{\partial d_1}{\partial C_1} &= \frac{\delta_2}{\beta_1\delta_2 - \beta_2\delta_1} \\ \frac{\partial d_1}{\partial C_2} &= \frac{\beta_2}{\beta_1\delta_2 - \beta_2\delta_1} \\ \frac{\partial C_1}{\partial A} &= -\frac{\lambda_0}{2\pi}\frac{1}{A} \\ \frac{\partial C_2}{\partial \phi} &= \frac{\lambda_0}{2\pi} \end{aligned} \tag{SI. 8}$$

and the standard deviations σ_A and σ_ϕ are determined from the distribution of experimentally retrieved values within a 5×5-pixel area as indicated by the white boxes in Fig. 6 in the main text.

References

(1) Henke, B. L.; Gullikson, E. M.; Davis, J. C. X-ray interactions: photoabsorption, scattering, transmission, and reflection at E= 50-30,000 eV, Z= 1-92. *Atomic data and nuclear data tables* **1993**, *54*, 181–342.
